# A Self-Forming Hydrogel from a Bactericidal Copolymer: Synthesis, Characterization, Biological Evaluations and Perspective Applications

**DOI:** 10.3390/ijms232315092

**Published:** 2022-12-01

**Authors:** Silvana Alfei, Alessia Zorzoli, Danilo Marimpietri, Guendalina Zuccari, Eleonora Russo, Debora Caviglia, Anna Maria Schito

**Affiliations:** 1 Department of Pharmacy, University of Genoa, Viale Cembrano, 16148 Genoa, Italy; 2 Cell Factory, IRCCS Istituto Giannina Gaslini, via Gerolamo Gaslini 5, 16147 Genoa, Italy; 3 Department of Surgical Sciences and Integrated Diagnostics (DISC), University of Genoa, Viale Benedetto XV, 6, 16132 Genova, Italy

**Keywords:** styrene-based bactericidal copolymer (CP1), dose-dependent cytotoxicity studies, human fibroblast, self-forming hydrogel, spectroscopic characterization, rheological experiments, swelling characteristics, porosity percentage, stability over time, kinetic mathematical models

## Abstract

Objects touched by patients and healthcare workers in hospitals may harbor pathogens, including multi-drug resistant (MDR) staphylococci, enterococci (VRE), *Escherichia coli*, *Acinetobacter*, and *Pseudomonas* species. Medical devices contaminated by these pathogens may also act as a source of severe and difficult-to-treat human infections, thus becoming a critical public health concern requiring urgent resolutions. To this end, we recently reported the bactericidal effects of a cationic copolymer (CP1). Here, aiming at developing a bactericidal formulation possibly to be used either for surfaces disinfection or to treat skin infections, CP1 was formulated as a hydrogel (CP1_1.1-Hgel). Importantly, even if not cross-linked, CP1 formed the gel upon simple dispersion in water, without requiring gelling agents or other additives which could be skin-incompatible or interfere with CP1 bactericidal effects in possible future topical applications. CP1_1.1-Hgel was characterized by attenuated-total-reflectance Fourier transform infrared (ATR-FTIR) and UV-Vis spectroscopy, as well as optic and scanning electron microscopy (OM and SEM) to investigate its chemical structure and morphology. Its stability was assessed by monitoring its inversion properties over time at room temperature, while its mechanical characteristics were assessed by rheological experiments. Dose-dependent cytotoxicity studies performed on human fibroblasts for 24 h with gel samples obtained by diluting CP_1.1-Hgel at properly selected concentrations established that the 3D network formation did not significantly affect the cytotoxic profile of CP1. Also, microbiologic investigations carried out on two-fold serial dilutions of CP1-gel confirmed the minimum inhibitory concentrations (MICs) previously reported for the not formulated CP1.Selectivity indices values up to 12 were estimated by the values of LD_50_ and MICs determined here on gel samples.

## 1. Introduction

The hospital environment and its objects, instruments, medical devices, and various surfaces handled by healthcare workers and/or patients are likely to become colonized with diverse groups of microbial agents [1,2]. Several different types of nosocomial infections are described, from skin infections to septicemias. Worryingly, about two million patients acquire nosocomial infection and at least 90,000 of them die, in the United States, every year [3,4]. Severe nosocomial infections are the fifth leading cause of death in critical-care hospitals. The prevalence of hospital-acquired infections (HAI) is attributable to the direct contact of healthy individuals with surfaces contaminated by bacteria and/or with patients becoming infected following direct contact with contaminated medical devices.

It has been reported that bacteria both of gram-positive and gram-negative species can survive for a variable duration of time, even up to months, on inanimate surfaces, including stethoscopes, white coats, adhesive tape, computer keyboards, elevator buttons, mobile communication devices, and ultrasound transducers [5,6,7]. 

The geographical position, the environmental conditions, as well as the presence of organic matter and the capability to form biofilms, strongly affect the rate of bacterial colonization and the survival of microorganisms [7,8].

While microorganisms such as *Streptococcus pneumoniae*, *S. pyogenes* and *Haemophilus influenzae* are fragile and display a short survival time on inanimate surfaces, nosocomial pathogens such as MRSA and MRSE, VRE, *E. coli*, *Pseudomonas* spp., and *Acinetobacter* spp. are more stable, thus becoming hazardous.

A case study by Katzenberger, Rösel, and Vonberg evaluated the survival kinetics of the most important nosocomial bacteria, including strains of *S. aureus*, *K. pneumoniae*, *P. aeruginosa*, *A. baumannii*, *S. marcescens*, *E. faecium*, *E. coli*, and *E. cloacae* on a panel of commonly used surfaces such as glass, polyvinyl chloride, stainless steel, or aluminum, at regular ambient temperatures and humidity [9]. According to the authors, the longest survival was observed for *A. baumannii* and *E. faecium* on all materials (at least four weeks), while *S. aureus* remained viable for at least one week. Among gram-negative species, *A. baumannii* demonstrated the longest survival, while other species were usually inactivated in less than two days [9]. Worryingly, nosocomial transmission of the above-mentioned bacteria may easily occur if no appropriate infection control measures are applied on a regular daily basis. Consequently, frequently touched objects in the hospital can represent a dangerous source of infections, mediating the transfer of the bacteria they harbor to the hands of healthcare workers, patients, and visitors, thus making the onset of infections, especially skin infections, highly probable.

Especially the latter type of infection can spread rapidly, both to the entire limb and through the bloodstream, causing serious damage that requires prompt treatment [10]. Adequate cleaning and disinfection procedures with specific bactericidal agents are a good solution to prevent and/or limit the transmission of pathogens from nosocomial surfaces to humans and the onset and progression of infections, including skin infections. However, effective new antibacterial agents are also needed to treat ongoing skin infections, which are often difficult to cure due to the high tendency of bacteria to develop resistance. Among bactericidal/antibacterial devices, those formulated as hydrogels could effectively satisfy both the need for powerful surfaces disinfectants and the urgent demand for new and effective antibacterial formulations suitable for topical administration. According to Oliver C. J. Andrén et al., prior to their study, most antimicrobial hydrogels were developed with the aim of disinfecting implants or surfaces in a hospital setting also by coating them. Unfortunately, such hydrogels were not suitable for directly coating tissue, mainly due to components incompatible with an in vivo environment [11]. Particularly, in their study, Andrèn et al. developed a non-toxic and hydrolytically rapidly degradable antibacterial hydrogel for the preventive treatment of surgical site infections during the first crucial 24-h period without relying on conventional antibiotics [11].

Currently, hydrogels with antibacterial components with few or no side effects compared to traditional ones have been developed in recent years as effective antibacterial agents [12]. Hydrogels consist of three-dimensional network structures obtained from synthetic and/or natural polymers capable of absorbing and retaining significant amounts of water [13,14]. Hydrogels obtained from several polymers have been produced and employed in tissue engineering, pharmaceutical, and biomedical fields [15]. The various preparation techniques adopted include physical cross-linking [16], chemical cross-linking [17], grafting polymerization [18], and radiation cross-linking [19,20]. In this regard, interest in physical or reversible gels has grown due to their relative ease of production and the advantage of not having to use cross-linking agents, thus avoiding the need for their removal before application. Anyway, particular techniques and the use of other additives are generally required to prepare physically crosslinked hydrogels, such as heating/cooling the polymer solution [21]; inducing ionic interaction by the adding of divalent or trivalent counterions [22]; triggering complex coacervation by mixing a polyanion with a polycation [23]; inducing the formation of intermolecular H-bonding by lowering the pH [24]; inducing aggregation by heat (maturation) [24]; or by freeze–thaw cycles [25]. Currently, by these techniques, advanced antibacterial hydrogels have been, and are being, developed, each with unique qualities, such as high-water swelling capacity, high oxygen permeability, improved biocompatibility, ease of drug loading and release, and structural diversity [26]. To limit the onset of resistance typically associated with conventional antibiotics that primarily affect the intracellular components of a bacterium, natural antimicrobial peptides (NAMPs), which act mainly through electrostatic forces damaging the bacterial membrane and rapidly killing pathogens [27], could be suggested for engineering antibacterial hydrogels. Unfortunately, several drawbacks including high production costs, mammalian cell toxicity, and low stability in vivo hinder their clinical application [27,28]. To overcome these problems, cationic antimicrobial polymers and dendrimers represent a strategic option to NAMPs because of their similar mechanisms of action, lower production prices, excellent antimicrobial effects, and low tendency to develop resistance [27,28]. In this regard, 3D porous materials (hydrogels) consisting of cationic polymer chains obtainable either by physical or chemical crosslinking [26] have been extensively studied as an alternative material for antibacterial applications. By carefully selecting monomers and crosslinkers, hydrogels that have desired capabilities, such as preferred hydrophilicity and porosity, can be developed and employed for antibacterial functions. In this scenario, we exploited two bactericidal cationic polymers (CP1 and OP2) [29], prepared in parallel with a one-component hydrogel made with only CP1, and a two-components hydrogel formulation containing both CP1 and OP2 as active ingredients, in which CP1 acted also as gelling agent. The study on the two-components hydrogel (CP1OP2-Hgel) has been recently published, along with the cytotoxic profile of the two not-formulated ingredients [30]. The formulation is currently under investigation concerning its antibacterial activity, in the hopes that it will have a synergistic effect. Here, we reported the synthesis and physicochemical characterization of the one-component CP1-based gel (namely CP1_1.1-Hgel) designed to avoid, in advance, possible antagonistic effects between CP1 and OP2. Particularly, CP1_1.1-Hgel was achieved by simply dispersing CP1 in water (1.1% wt/v) and was characterized by ATR-FTIR, UV-Vis spectroscopy, optic and scanning electron microscopy (OM and SEM) to investigate its chemical structure and morphology, while its mechanical properties were studied by rheological experiments. To complete the characterization of CP1_1.1-Hgel, experiments were conducted to determine the weight-lost profile over time and under gentle heating; the equilibrium swelling ratio percentage; the maximum swelling capacity; and porosity percentages. Additionally, the stability of a 3.0% wt/v CP1-gel was evaluated over four months at room temperature by monitoring its inversion properties. Although dose-dependent cytotoxicity experiments were previously carried out on human normal fibroblast with the not-formulated CP1 [30], and MICs of pristine CP1 have been recently reported against several MDR bacteria [29], here, dose-dependent cytotoxicity experiments with samples of CP1-based gel obtained by diluting CP1_1.1-Hgel in PBS were carried out in similar conditions to investigate if the formation of the 3D network affected the cytotoxic behavior of CP1. Similarly, new MICs were determined on serial two-fold dilutions of CP1_1.1-Hgel for the same reason.

## 2. Results and Discussion

Before presenting and discussing the results of this study, we thought it would be useful for readers to read a summary of the main characteristics, in terms of its physicochemical features and antibacterial/bactericidal activity, of the styrene-based copolymer CP1 used to prepare CP1_1.1-Hgel (Table 1).

### 2.1. Preparation of CP1_1.1-HGel

**Figure 1:**
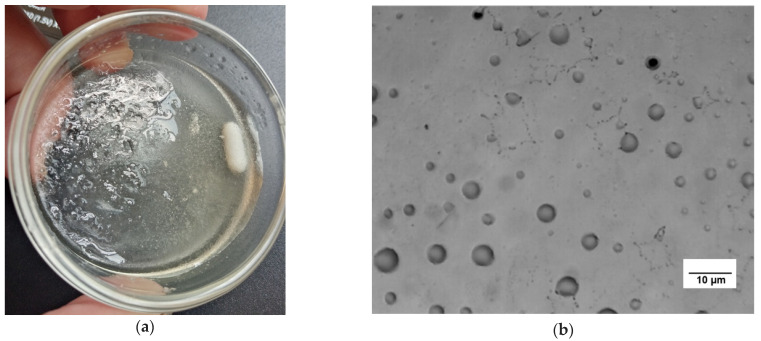
Petri dish (PD) containing CP1_1.1-Hgel obtained as described in Section 3.2 (**a**); optical microphotograph obtained analyzing hydrogel microparticles onto a glass surface (**b**).

After having prepared the two-components hydrogel CP1OP2-Hgel currently under investigation over its antibacterial activity, we decided to prepare a one-component hydrogel to avoid, in advance, possible antagonistic effects deriving by the simultaneous presence of both CP1 and OP2. Among CP1 and OP2, CP1 was selected because it showed MICs lower than those of OP2 (MICs = 0.1–0.8 µM) against several MDR clinical isolates, including the species mostly found in hospital settings [29]. In addition, CP1 was preferred to OP2, because, even though not cross-linked, it demonstrated the ability to form the hydrogel upon simple dispersion in water at a concentration lower than those used to prepare CP1OP2-Hgel, without the need of gelling agents or other additives, thus limiting possible skin-incompatibilities in future topical administrations. Also, by not employing additives, molecules that could interfere with the antibacterial effects of the original not-formulated CP1 would be avoided. Particularly, CP1_1.1-Hgel was obtained by dispersing CP1 in water (1.1% wt/v, 11 mg/mL) under stirring at room temperature, and then by degassing the obtained gel for 9 min to eliminate any air bubbles trapped in the 3D network of the gel. From the literature research, we found that antibacterial hydrogels, including cationic hydrogels, were prepared with different concentrations of antibacterial agents and other ingredients ranging from 4.6% to 30% in water or water/EtOH [31,32,33]. Our hydrogel was prepared at CP concentration 1.1% wt/v with water to obtain a gel with a consistency like that of commercial hand disinfectants on the market. As shown by the rheological experiments discussed later in this paper, the prepared CP1_1.1-Hgel was obtained as an elastic Bingham pseudoplastic semisolid formulation, modellable using the Herschel–Bulkley viscosity model, and owning a shear-thinning behavior, differently from the recently reported CP1OP2-gel which was demonstrated to be a shear thickening dilating fluid. In this regard, gels having a shear-thinning behavior typically allow an easy topical application, as they will be increasingly fluid during their spreading on the skin. Figure 1a shows the obtained hydrogel, while Figure 1b shows an optical microphotograph obtained by analyzing hydrogel microparticles on a glass surface with a phase contrast microscope at ×10 magnification.

### 2.2. UV-Vis Analyses

**Figure 2:**
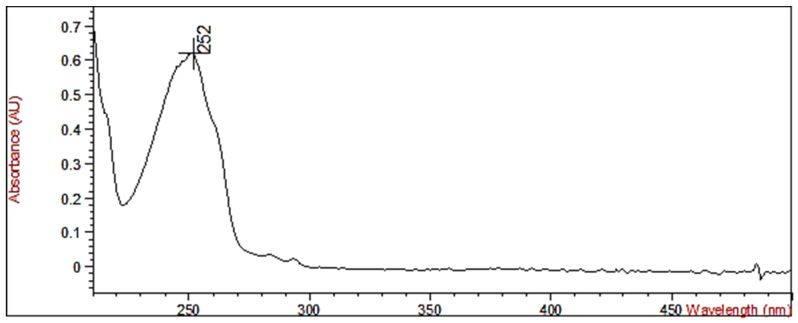
UV-Vis spectrum of CP1_1.1-Hgel.

As expected, the UV–Vis spectrum of CP1_1.1-Hgel at concentrations 50 µg/mL (MeOH), showed an absorption at λ_max_ = 252 nm, like that of the monomer M1 used to prepare the copolymer CP1, in turn used to obtain the gel (Figure 2) [29]. In this way it was established that during the formation of both copolymer and gel no interaction occurred that might affect the chromophore characteristics. 

### 2.3. ATR-FTIR Spectra

The analyses were made directly on samples of the soaked gel and of the dried film obtained by depositing the gel on a glass-slide and heating it until dried. 

**Figure 3:**
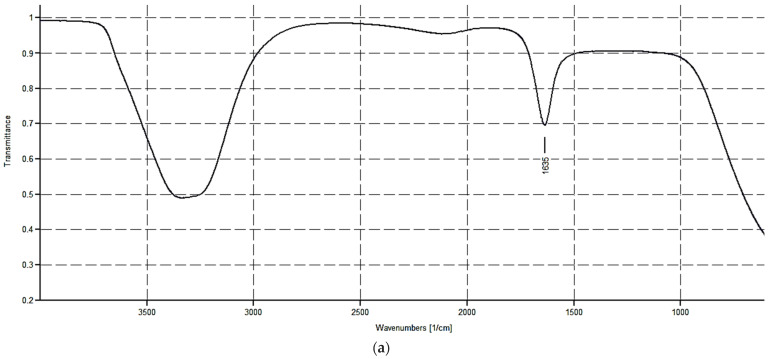

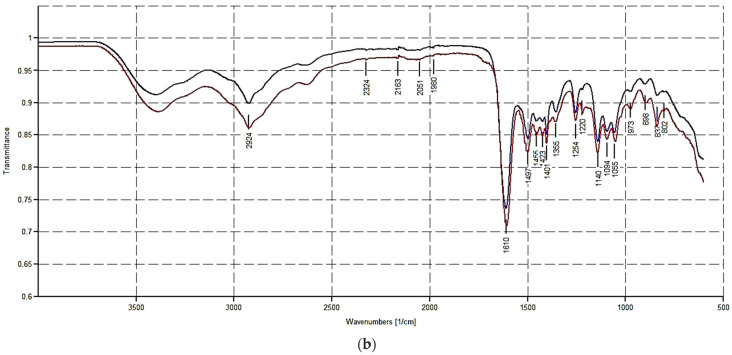
ATR-FTIR spectra of soaked CP1_1.1-Hgel (**a**) and of dried CP1_1.1-Hgel (black line) compared with the spectrum of CP1 (red line) (**b**).

The ATR-FTIR spectrum of the soaked CP1_1.1-Hgel revealed a very simple spectrum (Figure 3a), showing only the typical bands of water (a large OH stretching band over 3000 cm^−1^, a very weak combination band just over 2000 cm^−1^ and an OH scissoring band at 1635 cm^−1^). This result can be justified by the high content of the water which, in CP1_1.1-Hgel, is present at the 98.9% wt. with respect to the copolymer CP1, which is present at 1.1% wt. On the contrary, the spectrum of the dried sample obtained by heating the soaked gel (Figure 3b, black line), was very similar to that of copolymer CP1 (Figure 3b, red line) [29], thus establishing that the interactions that occurred during the formation of the gel did not affect the main functional groups of original CP1. Particularly, weak bands at 1497, 1401, 1254, and 1140 cm^−1^ provided by the aromatic C=C and C-N stretching bands, and intense bands at 1610 cm^−1^ standing for the C=O stretching of the amide group of DMAA, were detectable in both spectra.

#### Principal Components Analysis (PCA) of ATR-FTIR Data

**Figure 4:**
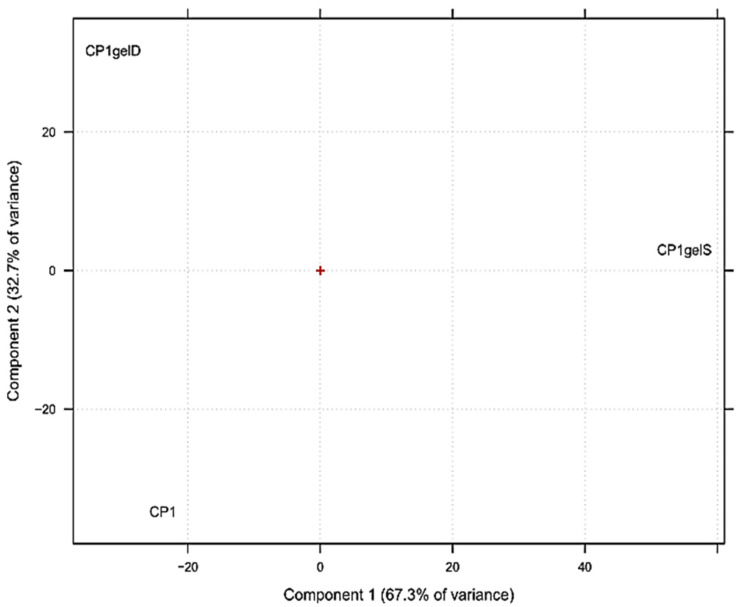
Results by PCAs performed on the matrix collecting spectral data of CP1, and of swollen and dried CP1-based gel, represented as a scores plot of PC1 vs. PC2. Red plus sign indicates score zero both on PC1 and on PC2.

First, a matrix of 10,203 variables was constructed collecting the spectral data (wavenumbers) of CP1, the swollen CP1-based gel, and the dried gel developed here. Then, the resulting dataset was first pre-treated by autoscaling and then processed using the PCA, which is an intriguing chemometric tool. PCA is capable of extracting the essential information from an intricate and enormous set of correlated variables by reducing them to a limited number of uncorrelated variables called principal components (PC) [34,35,36,37,38]. Among other possible plots, the PCA can allow for the visualization of the reciprocal positions occupied by the analyzed samples in the scores plot, where the scores are the coordinates of samples in the new orthogonal space of the new PCs. Particularly, the scores plot allows for the evaluation of the behavior of the samples in the new orthogonal space defined by the PCs, highlighting similarities and differences in the chemical composition of samples. Here, the scores plot of the three analyzed samples (PC1 vs. PC2) is shown in Figure 4.

As is observable, while on PC1 the samples were separated on the base of their water content, on PC2 the samples were separated depending on their structural characteristics. In particular, while on PC1, the swollen gel (CP1gelS) was located at very high positive scores, dried materials were both located at negative scores on the left of the plot, highlighting their very different water contents. Similarly, on PC2, while the gel materials (CP1gelS and CP1gelD) were both located at positive scores (even if very distant among them on PC1, due to their strongly different water contents), the original copolymer CP1 was located at negative scores (even if on the left side of plot as CP1gelD, evidencing their similar trivial water contents).

### 2.4. Scanning Electron Microscopy (SEM)

**Figure 5:**
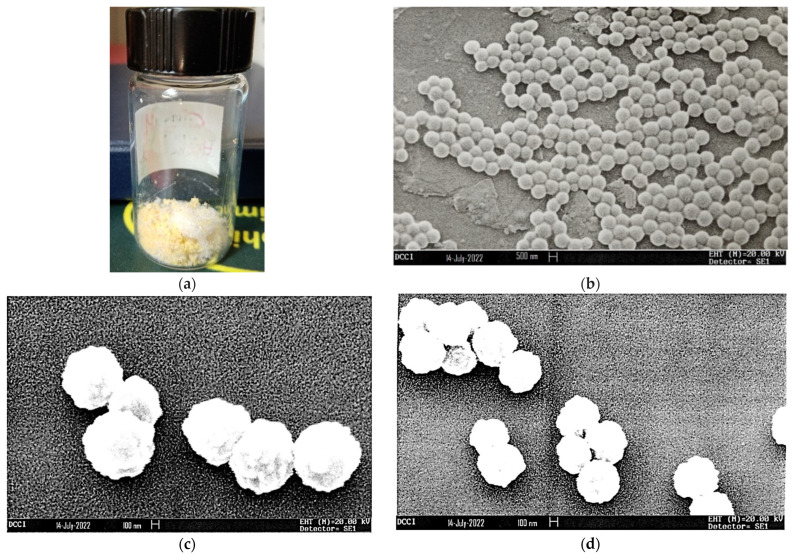
Lyophilized CP1_1.1-Hgel (**a**); representative SEM micrographs of a freeze-dried sample of CP1_1.1-Hgel (**b**–**d**).

The microstructure of the lyophilized hydrogel was investigated by SEM analysis. The aspect of the lyophilized CP1_1.1-Hgel is shown in Figure 5a, while SEM micrographs representative of all the obtained SEM images are shown in Figure 5b–d. The SEM image of the lyophilized cationic CP1_1.1-Hgel in Figure 5b revealed a spherical morphology, a narrow polydispersity, and apparently not porous particles with diameters of about a micron which tended to stick to each other. Magnifications in Figure 5c,d, evidenced that particles of lyophilized CP1-gel have an irregular and rugose surface and that the mean size estimable by particles shown in the two Figures is about 690 nm. Although rarely observed, the particles of CP1_1.1-Hgel shown in Figure 5b revealed a not-porous spherical morphology like those of poly-(MAA/EGDMA)/Fe_3_O_4_ hydrogel microspheres obtained by Park and colleagues and shown in Figure 2b of their article [39]. In particular, although other analyses have evidenced that by removing water from the CP1_1.1-Hgel the original CP1 was obtained, SEM analysis on lyophilized CP1-gel showed particles with a mean size lower than that determined for CP1 particles by dynamic light scattering (DLS) analyses previously reported (Table 1), since DLS provides a hydrodynamic diameter. In fact, the hydrodynamic diameter is generally larger than the real particle diameter because of the presence of ligands and solvated water molecules. 

### 2.5. Weight Loss (Water Loss) Percentage over Time

Figure S1a in Supplementary Materials (SM) shows the appearance of CP1_1.1-Hgel at time t_0_ of the experiments carried out to monitor weight loss over time when contained in the PD used to prepare it, as described in Section 3.2. Figure S1b shows the appearance of CP1_1.1-Hgel at the same time t_0_ when contained in wells (Ws) of the 24-wells plate. Differently, Figure S1c,d show the appearance of the fully dried samples when contained in PD and in Ws, respectively.

As observable in Figure S1c,d, when heat dried, CP1_1.1-Hgel leaves a thin film on both the glass and plastic surfaces. Due to this characteristic, CP1_1.1-Hgel could be used to reversibly coat various contaminated and/or contaminable surfaces in a hospital environment and inhibit or prevent their colonization by dangerous pathogens.

Table 2 collects the data of the experiments, including the weights of gels in Ws and in PD determined at times T_0_–T_x_.

**Figure 6:**
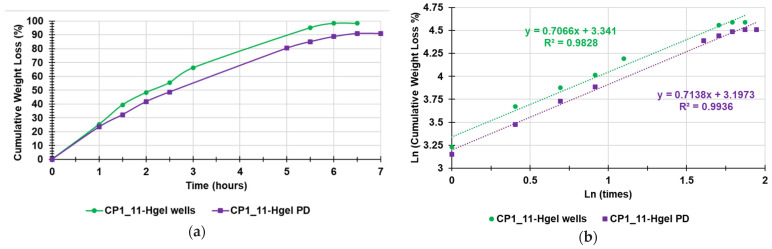
Cumulative weight loss percentage curves of the gel into Ws (green line) and of the gel contained in the PD used to prepared it and described in Section 3.2. (purple line) (**a**); Korsmeyer–Peppas kinetic models fitting data of cumulative weight loss curves (**b**).

Figure 6a shows the cumulative weight loss percentage curves of the gel into Ws (green line) and of the gel contained in the PD used to prepared it and described in Section 3.2. (purple line). Figure 6b shows with the same colors as the Korsmeyer–Peppas kinetic models fitting data of cumulative weight loss curves shown in Figure 6a.

As can be seen in Figure 6a, although a small amount of water was retained by the gel in the PD compared to that in the wells even for longer heating times, as established by performing an independent-samples *t*-test, also called a two-sample *t*-test, or Student’s *t*-test, no significant differences were observed between the two experiments, either in terms of water loss or in terms of the water release profile, and therefore of the kinetics that govern both releases. Based on these results, it can be assumed that the weight loss is not influenced neither by the initial amount of gel, nor by the material of the container. To exactly know the kinetics and the main mechanisms that govern the loss of water from CP1_1.1-Hgel, the data of both curves in Figure 6a were fit with the zero order model (% cumulative water release vs. time), first-order model (Log10 of % cumulative water remaining vs. time), Hixson–Crowell model (cube root of % cumulative water remaining vs. time), Higuchi model (% cumulative water release vs. square root of time), and Korsmeyer–Peppas model (Ln of % cumulative water release vs. Ln of time) obtaining the related dispersion graphs [40,41,42,43]. The highest value of the coefficient of determination (R^2^) of the equations of the linear regressions of these graphs was considered as the parameter for determining which model best fits the water release data. The R^2^ values were reported in Table 3 and established that the water loss for both the Ws and PD hydrogels best fitted the Korsmeyer–Peppas kinetic model (Figure 6b). 

According to what is reported in the literature the Korsmeyer–Peppas kinetic model is given by Equation (1):

Mt/M∞ = Ktn(1)

where Mt is the amount of water lost at time t, M∞ is the total amount of water released at infinite time, k is the release rate constant, and n is the release exponent indicating the type of release mechanism, which in the equations of the linear regressions in Figure 6b corresponds to the slopes [44]. In both cases, n ranged between 0.5 and 1, indicating a combination of different mechanisms, thus establishing that the loss of water by both systems was an anomalous diffusion (non-Fickian) [44].

### 2.6. Equilibrium Swelling Rate

**Figure 7:**
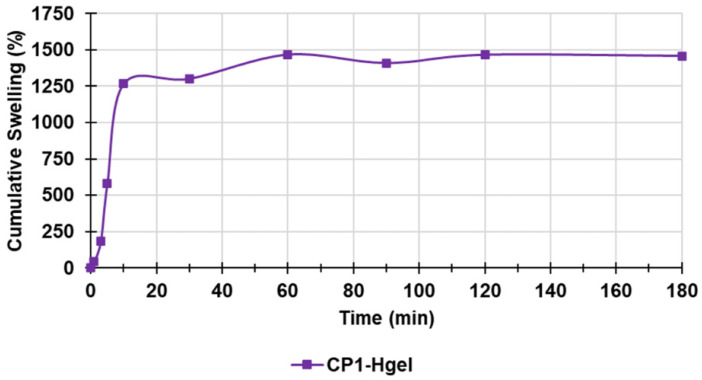
Cumulative swelling rate percentage curve of CP1_1.1-Hgel.

Swelling measurements were made at fixed times following the procedure described in Section 3.7, until the weight of swollen gel was approximately constant. Table S1 in SM collects the data of the experiments determined at times T_0_–T_9_, while Figure 7 shows the cumulative swelling rate percentage curve of gel.

The equilibrium swelling rate (Q_equil_), which was determined at the point where the hydrated gels reached a constant weight, was achieved after about 1 h of hydration, as reported in the study by Baron et al. [45] and was 1466%. Such value was higher than those of six out of eight hydrogels reported by Baron et al. [45].

### 2.7. Maximum Swelling Capacity (%) and Porosity (%) of CP1_1.1-Hgel

The maximum swelling capacity percentage of the CP1-gel by volume was determined by dispersing lyophilized samples of gel in an excess of water and after 9 min degassing, as reported in the experimental Section 3.9. Since from the SEM analyses, porosity and pores size of dried gel were not detectable, we calculated the porosity percentage of the swollen CP1-gel as previously reported [46], by the data obtained in these experiments. The determinations were made in triplicate and results were obtained by a means of three independent experiments ± standard deviation (SD). Figure S2 in SM shows the appearance of a swollen CP1_1.1-Hgel obtained by hydrating a sample of lyophilized gel (1 mL, 91.3 mg) with an excess of 11 mL of water, and recovered by centrifugation at 4000 rpm for 25 min. In this representative experiment, an initial volume of the lyophilized CP1-gel of 1 mL was increased up to 4 mL, establishing that the lyophilized gel has absorbed 3 mL of water, giving a sticky gel having a concentration of 30.3 mg/mL (3.0% wt/v). As reported [47], the stability over time of this CP1-gel was assessed evaluating its inversion properties after one, two, three, and four months from its first preparation, staying at room temperature. Figure S3a–e in SM, shows the appearance of a 1 mL of the 3.0% CP1-gel in the inverted position when just prepared, and after 1, 2, 3 and 4 months, respectively. As observable, the inversion properties of CP1-gel were maintained unchanged over the monitoring time, thus establishing a good stability of the hydrogel.

Table 4 reports the results of the maximum swelling capacity (S%) and porosity (%) obtained in this representative experiment.

As can be seen in Table 4, S (%) was 300% while P (%) was 75%, thus confirming a very high porosity and a remarkable capacity to absorb water, which in 9 min was two-fold higher than that observed for the hydrogel prepared by Siboro et al. after five days [48]. It has been reported that ideal wound-healing hydrogels should possess antibacterial properties, high porosity, and good swelling properties [49]. In this regard, since it possesses all three requirements, in addition to hypothesizing a future use of CP1_1.1-Hgel for surfaces disinfections and for the treatment skin infections, we could also hypothesize its possible future application in promoting wound healing. Curiously, the maximum swelling capacity obtained here (S = 300%) was significantly lower than the value of the equilibrium swelling rate (Q_equil_ = 1466%) determined in the previous Section 2.6. A first hypothesis to justify this fact, could be derived by considering that the dried materials used in the two experiments were of different origins: lyophilization in the experiment described here, and heating for 7 h in the experiment carried out in the previous Section 2.5. In this regard, it could be assumed that smaller pores were obtained in the lyophilization dried samples (as confirmed by SEM analyses from which porosity was not detected), while larger pores were obtained from heating the dried samples, favoring the swelling degree of CP1-hydrogel [45]. Additionally, while Q_equil_ is a cumulative swelling (%) reached over consecutive and cumulative additions of water at fixed times over 1 h, the maximum swelling percentage [S (%)] was reached upon a single addition of an excess of water followed by centrifugation.

### 2.8. Rheological Studies

**Figure 8:**
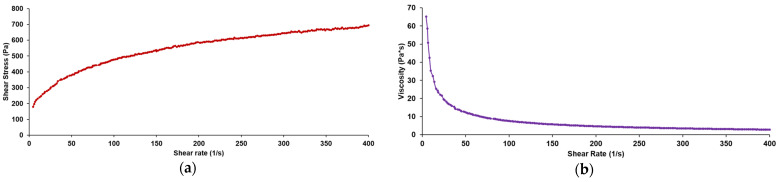
Curve of shear stress vs. shear rate (**a**) and viscosity vs. shear rate (**b**) of CP1_1.1-Hgel by rheological experiments (Pa*s in Figure 8b means Pa × s).

Rheology is a fundamental parameter to be considered when a formulation for skin application is prepared. From the data collected performing rheological studies on the prepared hydrogel, two types of graphs were constructed: one reporting the shear stress (τ [Pa]) as a function of the shear rate (γ. [s^−1^]) (Figure 8a), and another reporting the viscosity (η [Pa × s]) as a function of γ. (Figure 8b). 

**Figure 9:**
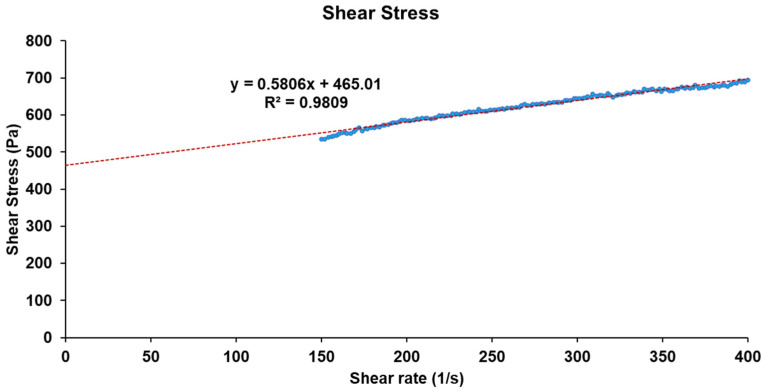
Linear tract of the curve of shear stress vs. shear rate (Bingham plastic behavior). Dynamic viscosity is a constant corresponding to the slope of the equation (0.5806), while the intercept (465.01) corresponds to the yield stress.

In Figure 8a, τ was not directly proportional to γ for values of γ < 150. Also, the tendency line of the dispersion graph is not linear, thus meaning that η was not constant, but decreases dramatically for small increases of γ (Figure 8b). On the contrary, for values of γ > 150, the tendency line of the dispersion graph becomes linear (Figure 9), meaning that η was practically constant, and did not change significantly also for greater increases of γ (Figure 8b). 

Collectively, CP1_1.1-Hgel is a non-Newtonian fluid, as other hydrogels previously prepared by us [50,51]. Note that for definition, a non-Newtonian fluid is a fluid that does not follow Newton’s law of viscosity given by equation Equation (2), where viscosity is constant and independent by the stress, and intercept is zero.

τ = η × γ(2)

For values of γ > 150, CP1_1.1-Hgel behaves as a Bingham plastic fluid, whose viscosity is constant, but intercept is >0. Particularly, in the equation of the linear tendency line associated to the dispersion graph of shear stress vs. shear rate, the viscosity of CP1_1.1-Hgel is represented by the slope (Figure 9), while the intercept corresponds to the yield stress, being the latter, the minimum stress required to make a material flow, which measures the strength of the material structure. Anyway, according to Figure 8a, CP1_1.1-Hgel shows a Bingham pseudoplastic behavior, in which shear stress and shear rate do not have a linear relationship. These fluids can be modeled using the Herschel-Bulkley viscosity model, and since η decreased as γ. increased, our sample should possess a shear-thinning behavior. 

For a semisolid formulation, the rapid decrease of η is a positive finding, because it allows us to apply the gel easily, as it will become increasingly fluid during its spreading on the skin.

For confirmation, the index flow (n) was determined by fitting the Herschel–Bulkley rheological model, which is expressed by Equation (3), to the data of shear stress vs. shear rate (Figure 8a).

τ = τoH + κHγnH(3)

where nH is the fluid flow behavior index, which indicates the tendency of a fluid to shear thin or thick, κH is the consistency coefficient, which serves as the viscosity indexes of the systems, and τoH is the Herschel–Bulkley yield stress point.

**Figure 10:**
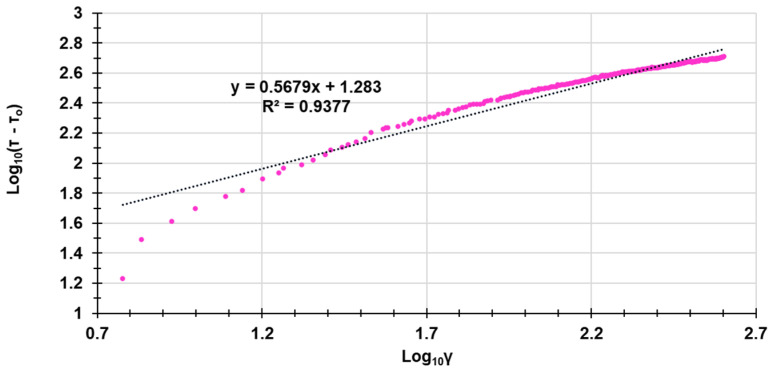
Hershel–Buckley rheological model.

According to the literature [52], the Hershel–Buckley rheogram is obtained by reporting in graph Log (τ − τo) vs. Log (γ) (Figure 10). 

In the plot, Log k and n were obtainable from the relative linear regression equation, where Log k is the intercept and n is the slope. The k value was calculated accordingly. 

The equation of the linear regression obtained by the Herschel–Buckley mathematical model, the coefficient of determination (R^2^), and the values of slope and intercept are reported in Table 5. 

Particularly, when n < 1, the fluid is shear thinning (pseudo-plastic fluid), when n = 1, the fluid is Newtonian, and when n > 1, the fluid is shear thickening (dilating hydrogels). Accordingly, the shear-thinning behavior previously assumed for CP1_1.1-Hgel, was confirmed, thus establishing that consistency of CP1_1.1-Hgel could allow for easy spreading on the skin by applying a light massage. Particularly, the shear thinning behavior is typical of relatively mobile fluids, such as weak gels and low-viscosity dispersions.

#### Frequency-Sweep Experiments

**Figure 11:**
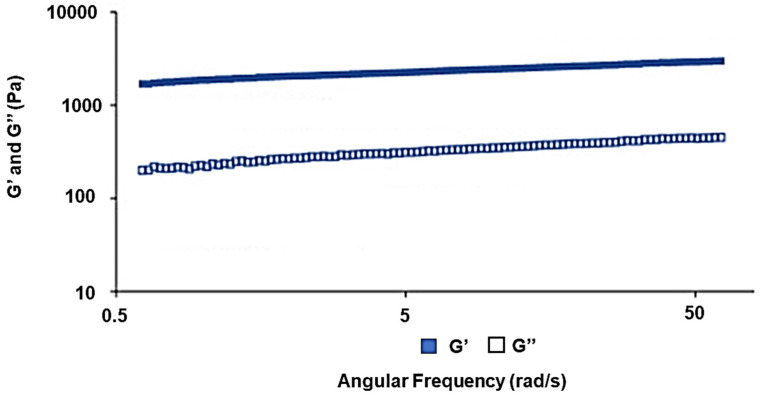
Elastic/storage modulus (G′) and viscous/loss modulus (G”) vs. angular frequency.

In frequency sweep tests, the elastic (G′) and viscous (G″) moduli were measured. Particularly, these parameters give information about the reversibly stored deformation energy (elastic behavior) and the irreversibly dissipated energy (viscous behavior) during one cycle, respectively [45]. As reported, hydrogels have a viscous behavior when the value of loss/viscous modulus (G″) is higher than that of storage/elastic modulus (G′), while have an elastic behavior if G′ > G″ [53]. In our case, from frequency-sweep experiments carried out as described in the experimental Section 3.10, the G′ values were always higher than the G″ ones and they are nearly independent on the angular frequency, thus suggesting that the network is formed and that CP1-based gel possesses an elastic behavior (Figure 11), similar to gels reported by Baron et al. [45].

Particularly, the magnitude of G′ was always much larger than that of G″ (Figure 11), thus establishing that the contribution of G″ to the magnitude of the complex modulus (G* = G′ + G″) was close to zero, and that G* tends to be equal to G′. These results imply that the physical behavior of CP1-based hydrogel is like that of a solid (elastic modulus).

### 2.9. Biological Evaluation of CP1-Based Hydrogel

#### 2.9.1. Antibacterial Effects of CP1-Based Hydrogel

Copolymer CP1 has been recently reported to have very low MIC values against several isolates of different species of MDR Gram-positive and gram-negative bacteria (MICs = 0.1 − 0.8 µM) [29]. Additionally, when tested by carrying out time-kill experiments on different isolates of the most clinically relevant gram-positive and gram-negative species, such as isolates of MRSA, *E. coli* and *P. aeruginosa*, CP1 demonstrated powerful and rapid bactericidal effects at concentrations 4 × MICs [29]. Here, by its simple dispersion in deionized water, without the use of other additives, such as stabilizing or gelling agents, CP1 was formulated as a hydrogel (CP1_1.1-Hgel). The antibacterial effects of two-fold serial dilutions of CP1_1.1-Hgel (1.1% wt/v, 70 µM) prepared to have concentrations of CP1 in the range 0.006–0.8 µM were investigated on the same bacteria used to assess the MICs of the not formulated CP1 [29], to evaluate if the 3D network formation would have influenced its antibacterial effects. Table 6 reports the MICs of CP1, the results obtained with CP1_1.1-Hgel, and the MICs of reference antibiotics. 

Rationally, since CP1_1.1-Hgel was composed only of CP1 and water, CP1-gels having CP1 concentrations as the MICs observed for not formulated CP1 should equally have inhibited the growth of the bacteria. As expected, the solutions of CP1-based gel inhibited the bacterial growth at the same concentrations of the not formulated CP1, thus proving that the interactions occurred to form the gel did not influence the antibacterial effects of CP1. For extension of these results, we are confident that also the bactericidal effects of CP1 would be kept in its gel formulation. 

#### 2.9.2. Dose-Dependent Cytotoxicity Experiments with CP1-Based Hydrogel

**Figure 12:**
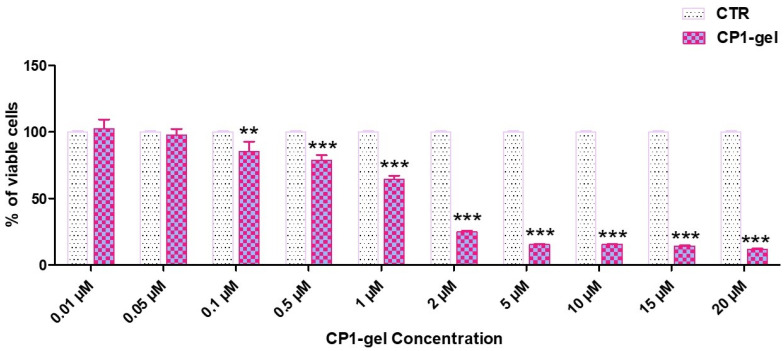
Results from dose-dependent cytotoxicity experiments carried out on MAIL-2 human cells at 24 h exposure to CP1-gel (CP1-gel concentration means the concentrations of CP1 in the gel samples used in the experiments). ** = *p* < 0.01; *** = *p* < 0.001 by two-way ANOVA statistical analyses.

Hypothesizing also a possible cutaneous use of CP1-based gel, both to treat skin infections and, due to its high porosity and swelling capacity percentages, to promote tissue regeneration and wound healing, its dose-dependent cytotoxicity was evaluated on human fibroblast (MAIL-2) at 24 h of exposure. We recently reported the dose-dependent cytotoxicity on MAIL-2 of not-formulated CP1 powder dissolved in PBS [30]. Here, as for the microbiologic tests, new dose-dependent cytotoxicity experiments were carried out with CP1-based gel to assess if the 3D network formation had affected the cytotoxic behavior of not-formulated CP1. To this end, sample of gels with concentrations of CP1 in the range 0.01–20 µM, were prepared by opportunely diluting CP1_1.1-Hgel in PBS. Subsequently, the LD_50_ of CP1-based gel and its selectivity indices (SIs) were determined. Notably, SIs measure the capability of a new antibacterial agent to selectively inhibit the bacterial cell without damaging the eukaryotic one. The SI values are given by the ratio between the concentration of the antibacterial agent capable of killing 50% of eukaryotic cells (LD_50_) and the values of MICs for a specific bacterium. Human fibroblasts were selected for cytotoxicity experiments because they are widely used for the evaluation of the cytotoxicity of compounds potentially finalized for topical administration as cosmetic formulations [54,55]. The concentrations ranging from 0.01 to 20 µM were chosen based on the very low MICs observed for CP1-based gel and reported in the third column of Table 6. The cells viability percentages as function of the concentrations of CP1 in the gel samples are shown in Figure 12. CTR bars are representative of samples of fibroblasts not treated with CP1-gel.

Cell viability greater than 50% was observable for CP1-gel samples with CP1 concentrations <2 μM, while at CP1 concentrations ≥2 μM, CP1-gel was found to be highly cytotoxic. Anyway, to get more realistic information on the feasibility of the clinical application of CP1-based gel, we determined its SIs against the clinically relevant MDR pathogens reported in Table 6, using the equation reported in the experimental Section 3.12. 

**Figure 13:**
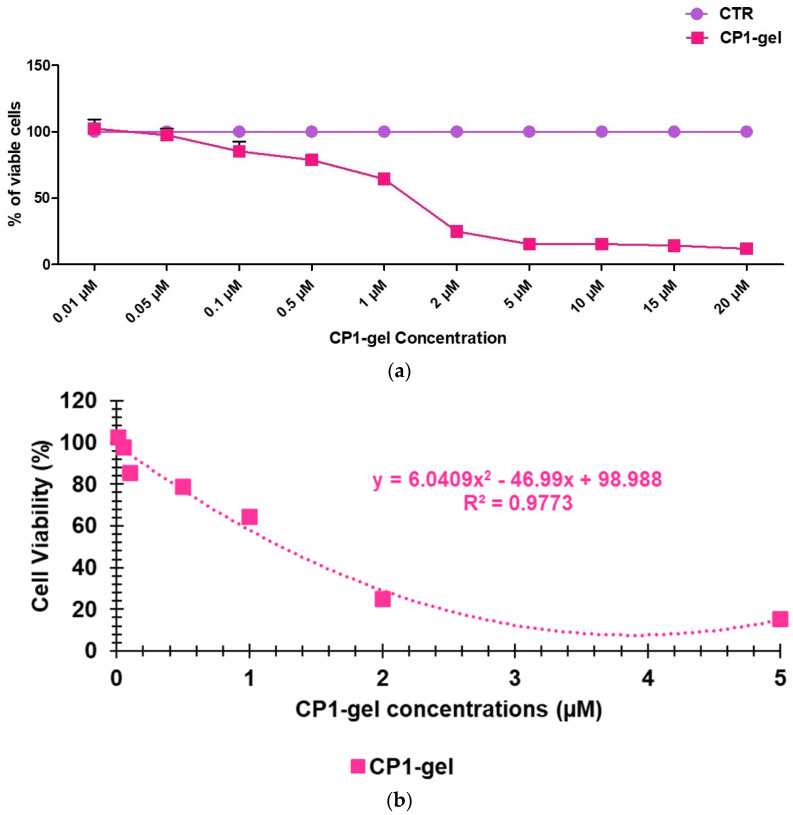
Curves of cells viability (%) of MAIL-2 cells exposed for 24 h to CP1-gels at concentrations of CP1 0.01–20 µM and of MAIL-2 cells not exposed to CP1-gels (CTR) (**a**); polynomial tendency line fitting the dispersion graph made with points in Figure 13a up to concentration 5 µM (**b**).

Particularly, to calculate the desired SI values, the value of LD_50_ of CP1-based gel was necessary. To this end, we plotted the data of cell viability % obtained at 24 h of exposure vs. the concentrations of CP1 in the samples of CP1-gels used, thus obtaining the curve shown in Figure 13a. Figure 13a shows also the curve of control (CTR) and CP1-gel concentration means the concentrations of CP1 in the gel samples used in the experiments.

Using the points of the curve in Figure 13a up to concentration 5 μM, because over such concentrations cell viability remained unchanged, the correspondent dispersion graph was obtained, and the best fitting tendency line was attained using the tool provided by Microsoft excel software. The tendency line was polynomial, its good fitting with the dispersion graph was assured by the very high value of the coefficient of determination (R^2^), and the equation of the tendency line was used to determine the desired LD_50_. Figure 13b shows the used dispersion graph, the best fitting tendency line, and the related equation used to compute the LD_50_ of CP1-gel. The obtained equation, the R^2^ value, the computed values of LD_50_ and the range of SI obtained for CP1-gel are reported in Table 7.

The SI values of CP1-gel computed for each isolate used to determine the MICs of CP1-gel are instead reported in Table 6.

According to Table 7 the LD_50_ of CP1-based gel was = 1.2 µM like that observed for the not formulated CP1, while the range of SIs was 1.5–12 against isolates of gram-positive species and 1.5–3 against strains of gram-negative family. These values are considered acceptable to hypothesize a new antibacterial agent as clinically applicable [56,57,58,59].

## 3. Materials and Methods

### 3.1. Chemicals and Instruments

**Scheme 1:**
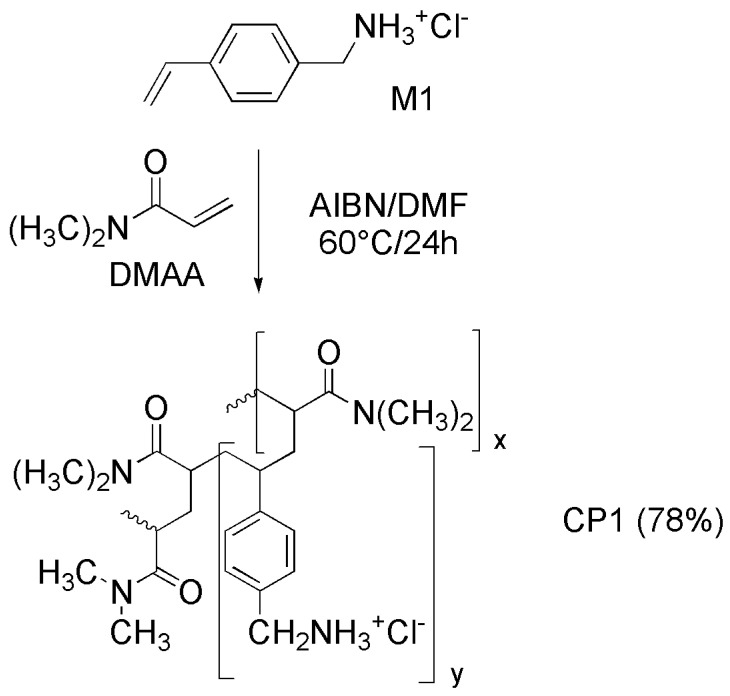
Synthetic route to prepare copolymer CP1 [29].

The styrene-based cationic monomer M1 necessary to prepare copolymer CP1 used in this work to form CP1_1.1-Hgel and CP1 were prepared according to procedures recently described [29]. Particularly, CP1 has been prepared by the radical solution copolymerization of M1 with dimethylacrylamide (DMAA) as shown in Scheme 1.

All reagents and solvents were from Merck (formerly Sigma-Aldrich, Darmstadt, Germany) and were purified by standard procedures. Azo-*bis*-isobutyronitrile (AIBN) used to reproduce the copolymerization reaction and prepare CP1 was crystallized from methanol (MeOH). Anhydrous magnesium sulphate (MgSO_4_) was used to dry organic solutions, which were evaporated by the mean of a rotatory evaporator at a reduced pressure (10–20 mmHg). The melting ranges of the solid compounds, the ATR-FTIR analyses, UV-Vis spectra, and scanning electron microscopy (SEM) images were obtained with the same instruments described in our previous article [29]. Silica gel (70–230 mesh) used to perform column chromatography was bought by Merck, (Washington, DC, USA). For the rheological characterization of the gel, it was utilized the concentric cylinder viscometer described in a study by Zuccari et al. [60]. Aluminum-backed silica gel plates (DC-Alufolien Kieselgel 60 F254), purchased by Merck (Washington, DC, USA), were employed to perform thin layer chromatography (TLC). Detection of spots was made by UV light (254 nm), utilizing a Handheld UV Lamp, LW/SW, 6W, UVGL-58 (Science Company^®^, Lakewood, CO, USA).

### 3.2. Preparation of CP1_1.1-HGel 

To prepare the CP1-based hydrogel, a procedure different from that used to prepare the CP1OP2-gel was adopted. First, we evaluated with preliminary experiments, the volume of water absorbed by CP1, which allowed to obtain a gel with a consistency like that of the commercial hand’s disinfectants on the market. Secondly, we weighted a quantity of CP1 which would have absorbed about 5 mL of water to obtain a quantity of gel sufficient to complete its characterization. Particularly, CP1 (55.2 mg; 0.35 µmol) was weighted in a Petri dish (PD) (Ø = 5 cm; h = 1.5 cm) and added at room temperature and under gentle magnetic stirring with deionized water, until a gel of a consistency like that of commercial hands disinfectants was obtained (5 mL). The concentration of the so obtained CP1-gel was 11 mg/mL. According to the molecular weight of CP1 a hydrogel containing CP1 at concentration 70.1 µM and at 1.1% wt/v CP1/water was obtained. The so prepared hydrogel (CP1_1.1-Hgel) was degassed for 9 min at room temperature using an Ultrasonic Cleaner 220 V, working at a frequency of 35 kHz, timer range 1–99 min, temperature range 20 to 69 °C (68 to 156 °F) (VWR, Milan, Italy). CP1_1.1-Hgel was then left in the Petri dish carefully sealed off to prevent water evaporation and kept in the fridge for subsequent characterization experiments, including UV-Vis, ATR-FTIR, optical microscopy, water loss, rheological studies, MICs determinations and dose-dependent cytotoxicity experiments. Additionally, a fraction of the prepared CP1_1.1-Hgel was lyophilized to complete its characterization by carrying out scanning electron microscopy (SEM) analyses, and by determining the maximum swelling capacity and porosity percentage. The swollen gel so obtained was subsequently monitored concerning its inversion properties over time at room temperature to assess its stability.

### 3.3. UV-Vis Analyses

To acquire the UV–Vis spectra of CP1_1.1-Hgel, it was diluted in MeOH until a gel concentration of 50 µg/mL (MeOH), and the ultraviolet profile of CP1_1.1-Hgel was detected in the range 210–500 nm. The max absorbance (Abs = 0.6192) was observed at λ_max_ = 252 nm (ε = 52,030). Acquisitions were made in triplicate, and the spectrum reported in Section 2.2, is a representative image.

### 3.4. ATR-FTIR Spectra

ATR-FTIR analyses were carried out both on the soaked gel and on the dry gel and directly on the samples. The spectra were acquired from 4000 to 600 cm^−1^, with 1 cm^−1^ spectral resolution, co-adding 32 interferograms, with a measurement accuracy in the frequency data at each measured point of 0.01 cm^−1^, due to the internal laser reference of the instrument. Acquisitions were made in triplicate, and the spectra showed in Section 2.3. are the most representative images. The spectral data obtained were then included with those of CP1 [29] in a dataset matrix and were processed using the principal components analysis (PCA), by means of CAT statistical software, (Chemometric Agile Tool, free down-loadable online, at: http://www.gruppochemiometria.it/index.php/software/19-download-the-r-based-chemometric-software; accessed on 25 November 2022). In particular, we arranged the FTIR data of the six spectra in a matrix 3401 × 3 (n = 10203) of measurable variables. For each sample, the variables consisted of the values of transmittance (%) associated to the wavenumbers (3401) in the range 4000–600 cm^−1^. The spectral data in the matrix were pretreated by autoscaling.

### 3.5. Optical Microscopy Analyses

Optical microphotographs of CP1_1.1-Hgel were obtained at ×10 magnification with the Olympus IMT-2 phase contrast microscope. CP1_1.1-Hgel was deposited on an optical microscope slide using a squeezable pipette made of polyethylene material (VWR, Milan, Italy), and then observed.

A representative microphotograph has been reported in Section 2.1.

### 3.6. Scanning Electron Microscopy (SEM)

The microstructure of the lyophilized hydrogel was investigated by SEM analysis. In the performed experiments, the hydrogel was frozen by fast immersion into liquid nitrogen. Later, the sample was lyophilized at −55 °C for 24 h, using a freeze–dry system (Labconco, Kansas City, MI, USA). Then it was broken in liquid nitrogen, fixed on aluminum pin stubs and sputter-coated with a gold layer of 30 mA for 1 min, to improve the conductivity and an accelerating voltage of 20 kV was used for the sample’s examination. The micrographs were recorded digitally using a DISS 5 digital image acquisition system (Point Electronic GmbH, Halle, Germany). 

### 3.7. Weight Loss (Water Loss) Percentage over Time

Samples of CP1_1.1-Hgel were taken with a truncated Pasteur pipette and deposited in three wells (B4, B5 and B6) of a plastic 24-wells plate in order to cover the bottom with a thin layer. The deposited quantities were weighed individually, and the total of the deposited gel was calculated (586.8 mg). At the same time, the glass PD utilized for preparing CP1_1.1-Hgel and containing a fraction of gel was weighed and the amount of the gel content was computed. The plastic plate and the glass PD were then placed in an oven under controlled temperature (37 °C) and weight loss (meaning water loss) was monitored as a function of time until a constant weight was reached (6.5 and 7 h respectively). The cumulative weight loss percentage was determined by means of the equation Equation (4):

(4) $$Weight\;loss\;\left(\%\right)=\frac{MQ-Mt}{MQ}\times 100$$

where *MQ* and *Mt* are the initial hydrogel mass and hydrogel mass after a time *t*, respectively.

### 3.8. Swelling Rate Percentage over Time

The swelling rate measurements were carried out immersing 171.3 mg of the dried hydrogel in the PD obtained in the previous experiment in deionized water in a test tube. At intervals of time selected according to the literature [61], the sample in the test tube was centrifugated (10 min, 4000 rpm) to remove the not absorbed water, inverted on filter paper to absorb residual water and weighted. The cumulative swelling ratio percentage (*Q*%) as a function of time was calculated from Equation (5):

(5) $$Q\;\left(\%\right)=\frac{WSt-WD}{WD}\times 100$$

where *WD* and *WSt* are the weights of the lyophilized gel and of the swollen gel at time *t*, respectively. The equilibrium swelling ratio (*Q*_equil_) was determined at the point (time *t*) the hydrated gels achieved a constant weight.

### 3.9. Maximum Swelling Capacity (%) and Porosity (%) of CP1_1.1-Hgel

In a graduated centrifuge tube to the tenth (Ø_est_ = 14 mm), a volume of lyophilized gel equal to 1 mL (Vi) was introduced to which excess water was gradually added (11 mL). The gel was gently shaken with a spatula for a few minutes to push away the trapped air and then degassed for additional 9 min at room temperature using an Ultrasonic Cleaner 220 V (VWR, Milan, Italy). Upon centrifugation at 4000 rpm for 25 min, the volume of the swelled hydrogel was measured (Vf). The values of Vi and Vf were used to calculate the percentage porosity (P%) and the percentage swelling (S%) according to the following Equations (6) and (7).

P (%) = 100 × (Vf − Vi)/Vf(6)

S (%) = 100 × (Vf − Vi)/Vi(7)

#### Stability of the CP1-Gel Obtained at its Maximum Swelling Capacity

The stability of CP1-gel at concentration 3.0% wt/v was assessed monitoring its inversion properties after one, two, three and four months from its first preparation staying at room temperature.

### 3.10. Rheological Studies

For the rheological characterization of CP1_1.1-Hgel, a concentric cylinder viscometer (Phisica Haake Thermo Fisher Scientific Inc., Waltham, MA, USA) equipped with a Z4 probe was used. For the determinations of dynamic viscosity, approximately 5 mL of hydrogel was placed in the viscometer and thermally equilibrated at the test temperature (25 ± 0.1 °C) for 60 min before the measurement; then, the sample was subjected to an increasing shear rate from 0 to 400 s^−1^ by a 100 s^−1^/min gradient. Additionally, by frequency-sweep experiments, oscillatory shear responses, such as G′ (elastic/storage modulus), and G″ (loss/viscous modulus) were determined over the angular frequency range 0.5–50 rad/s, by varying the frequency between 0.1 Hz to 10 Hz, with a fixed 1% strain and 37 °C, as temperature. All the experiments were performed in triplicate and mean values were used in analysis.

### 3.11. Antibacterial Activity and Dose-Dependent Cytotoxicity Experiments

#### 3.11.1. Microorganisms

The 28 strains of different species of MDR Gram-positive and Gram-negative bacteria reported in a previous study by us [29] were here utilized to assess the antibacterial effects of CP1_1.1-Hgel. All were clinical isolates from human specimens and were identified by matrix-assisted laser desorption/ionization time-of-flight (MALDI-TOF) mass spectrometric technique (Biomerieux, Firenze, Italy) or by VITEK^®^ 2 (Biomerieux, Firenze, Italy).

#### 3.11.2. Determination of the Minimal Inhibitory Concentrations (MICs) of CP1-Based Gel

The antibacterial effects of CP1_1.1-Hgel on the 28 pathogens previously described, were assessed by determining their MICs following the microdilution procedures detailed by the European Committee on Antimicrobial Susceptibility Testing (EUCAST) [62]. 

Briefly, as already reported [63], overnight cultures of bacteria were diluted to obtain a standardized inoculum of 1.5 × 10^8^ CFU/mL. Appropriate aliquots of each suspension were added to 96-well microplates containing the same volumes of serial 2-fold dilutions (CP1 concentration in gels ranging from 0.8 to 0.006 μM) of CP1_1.1-Hgel, to achieve a final concentration of about 5 × 10^5^ cells/mL. The plates were incubated at 37 °C for 24 h and then the MICs were read observing where in the 96-well microplates the bacteria growth was inhibited. Particularly, the concentration of the first well in the series of wells at decreasing concentrations of the samples, where no bacterial growth was observed, was recorded as the MIC. Medium not containing the tested substance was used as control. All MICs were obtained at least in triplicate, and results were expressed reporting the modal value. In case of equivocal or not clear results more than three determinations of MICs were carried out.

#### 3.11.3. Human Fibroblasts Isolation and Culture

Human primary fibroblasts were isolated and cultured as previously reported [30].

#### 3.11.4. Viability Assay

Fibroblasts were thawed, expanded, and seeded in 96-well plates (at 4 × 10^3^ cells/well) in complete medium for 24 h. The seeding medium was removed and replaced with fresh complete medium supplemented with CP1_1.1-Hgel at increasing concentrations of CP1 (0 μM, 0.01 μM, 0.05 μM, 0.1 μM, 0.5 μM, 1 μM, 2 μM, 5 μM, 10 μM,15 μM, 20 μM). Cells (quadruplicate samples for each condition) were then incubated for additional 24 h. The effect on cell growth was evaluated by a fluorescence-based proliferation and cytotoxicity assay (CyQUANT^®^ Direct Cell Proliferation Assay (Thermo Fisher Scientific, Life Technologies, Milano Brianza (MB), Italy) according to the manufacturer’s instructions. Briefly, at each time points, an equal volume of detection reagent was added to each well and incubated for 60 min at 37 °C. The fluorescence signal was measured using the monochromator based M200 plate reader (Tecan, Männedorf, Switzerland) set at 480/535 nm. 

### 3.12. Selectivity Indices (SIs) Determination

First, from the experiments described in previous Section, we determined the LD_50_.values at 24 h of exposure of MAIL-2 cell to CP1-gels. The obtained LD_50_ and the MIC values determined for CP1-based gels were used to calculate the SI values of CP1_1.1-Hgel against each isolate on which it was tested according to equation Equation (8):

SI = LD_50_/MIC (8)

where LD_50_ is the dose of CP1_1.1-Hgel which was capable to halve the cell viability percentage at 4 h of exposure, while MIC is the minimal dose of CP1_1.1-Hgel capable to inhibit the bacteria growth.

### 3.13. Statistical Analyses

All the experiments were performed at least in triplicate. The results from cell viability studies were expressed as mean ± SD (standard deviation). A two-way analysis of variance (ANOVA) with the Bonferroni correction was employed for determining the statistical significance of differences between experimental and control groups. Prism 5 software (GraphPad, La Jolla, CA, USA) was used to analyze and present data and asterisks in the images indicate the following p-value ranges: ** = *p* < 0.01, *** = *p* < 0.001.

## 4. Conclusions

Here, we have prepared and completely characterized a new hydrogel using the recently reported bactericidal styrene-based copolymer CP1. Thanks to the self-forming gel capability of CP1, the CP1_1.1-Hgel developed here was obtained by an operator-friendly, low-cost, and scalable procedure. No additional ingredient was employed, which may affect the biological properties of CP1 or may be dangerous or skin-incompatible in possible future in vivo administrations. The UV-Vis analysis of the soaked CP1_1.1-Hgel proved that the interactions occurred for forming the gel network did not affect the chromophore characteristics of CP1. Additionally, the ATR-FTIR spectra acquired on a dried sample of gel, established that such interactions are reversible. Weight loss experiments showed that the release of water contained in the gel is almost quantitative, leaves thin films on surfaces, and follows the Korsmeyer–Peppas kinetics, which are governed by non-Fickian anomalous diffusion. Experiments to find the equilibrium swelling ratio percentage on dried samples of gel, demonstrated that dried samples reform the hydrogel swelling of 1466% in 60 min. Experiments to determine the porosity percentage and the maximum swelling capacity by volumes on lyophilized gel, showed very high porosity (%) and swelling (%). Stability studies over a period of four months by monitoring the inversion properties of a 3.0% wt/v CP1-gel showed unchanged characteristics and optimal stability at room temperature. Rheological studies showed that CP1_1.1-Hgel is an elastic Bingham pseudoplastic fluid following the Herschel–Bulkley viscosity model, and possessing a shear-thinning behavior, thus evidencing an easy spreadability if applied for a cutaneous use. Finally, microbiologic experiments on several MDR bacteria and dose-dependent cytotoxicity studies carried out on MAIL-2 cells, with samples of gel obtained diluting CP1_1.1-Hgel, provided results matching those obtained with not formulated CP1, thus establishing that the 3D network formation did not influence the pharmacological properties of CP1. Interestingly, SI values up to 12 were estimated against the most common and clinically relevant MDR bacteria harboring nosocomial surface and medical devices and transmittable to humans with dramatic consequences. Collectively, although far more work and more in-depth investigations are necessary to confirm the actual clinical applicability of the CP1-gel developed here, due to its powerful bactericidal effects, its favorable selectivity indices against several MDR-pathogens, its good mechanical properties, stability, high porosity percentage, and easy spreadability, CP1_1.1-Hgel may in future being used both for the disinfection of hospital surfaces, and for treating severe nosocomial skin infections by topical administration. Additionally, due to its high porosity percentage and its high capability to absorb liquids as wounds exudate, CP1-based hydrogel may be used also as an antibacterial wound-healing agent. 

## Figures and Tables

**Table 1:** Characterization data of CP1 here used as single ingredient to prepare CP1_1.1-Hgel.

Analyses	Data		
**ATR-FTIR**	**Bands (cm^−1^)**	**Groups**	
	3500–3000	(NH_3_^+^)	
	2992	(CH_2_)	
	1607	C=O	
	838	CH bending phenyl 1,4-disubstituted	
**DLS**	Z ave (nm)	833.4 ± 10.1	
	Z potential (ζ-p (mV))	0.235 ± 0.022	
	PDI	+27.3 ± 4.2	
**Viscosity**	Inherent viscosity (η_inh_):	1.22 ± 0.02 dL/g	
	Intrinsic Viscosity [η]	1.68 ± 0.02 dL/g	
	Relative Viscosity (η_rel_)	1.84 ± 0.02 dL/g	
	Specific Viscosity (η_sp_)	0.84 ± 0.02 dL/g	
**Molecular Weight**	Mr	157,306	
**NH_2_ Equivalents Content**	Volumetric Titration	30.98 ± 0.13 mequiv._NH2_/g	
	Potentiometric titration	31.37 ± 0.08 mequiv._NH2_/g	
**Potentiometric Titrations**	End Point	pH = 3.5	
		V added (mL) = 8	
		Species	MICs range (µM)
**Microbiology**	Minimum Inhibitory Concentratios (MICs)	Enterococci	0.4–0.8
		Staphylococci	0.1–0.8
		*Enterobacteriaceae*	0.4–0.8
		Non-fermenting species	0.4–0.8
		Strains	Max Log_10_ reduction (time)
	Time killing	*S. aureus*	>3 (1 h)
		*P. aeruginosa* 1V	5 (2 h)
		*P. aeruginosa* CR	5 (2 h)
		*E. coli*	4 (4 h)

**Table 2:** Weights of gels in Ws and in the PD determined at times T_0_–T_x_.

Time * (min)		B4 (mg)	B5 (mg)	B6 (mg)	Ws (mg)	Weight Loss (%) *	Time ** (min)		PD (g)	Weight Loss (%) **
T_0_	0	224.0	181.7	181.1	586.8	0	T_0_	0	2.8954	0
T_1_	60				438.1	25.3	T_1_	60	2.2177	23.4
T_2_	90				355.5	39.4	T_2_	90	1.9591	32.3
T_3_	120				303.2	48.3	T_3_	120	1.6867	41.7
T_4_	150				261.9	55.4	T_4_	150	1.4866	48.7
T_5_	180				198.6	66.2	T_5_	300	0.5662	80.44
T_6_	330				28.4	95.2	T_6_	330	0.4310	85.1
T_7_	360				10.2	98.3	T_7_	360	0.3235	88.8
T_8_	390				10.2	98.3	T_8_	390	0.2620	91.0
							T_9_	420	0.2615	91.0

* Referred to the gel in the plastic Ws; ** referred to the gel in the glass PD.

**Table 3:** Values of coefficients of determination obtained for all the kinetic models used.

Kinetic Model	R^2^ Hydrogel in Ws	R^2^ Hydrogel in PD
Zero order	0.9525	0.9667
First Order	0.9549	0.9906
Hixson-Crowel	0.9525	0.9667
Higuchi	0.9780	0.9777
**Korsmeyer-peppas**	**0.9828**	**0.9936**

**Table 4:** Results from swelling experiments.

Sample	Vi (mL)	Weight (mg)	Vf (mL)	P (%)	S (%)
CP1_1.1-Hgel	1	91.3	4	75	300

**Table 5:** Equation, coefficient of determination (R^2^), slope and intercept values of the linear regressions obtained by fitting the rheogram’s data to the Herschel–Buckley mathematical model.

Mathematical Model	Equation	R^2^	Slope (n)	Intercept (LogK)	K
Herschel-Buckley	y = 0.5679x + 1.283	0.9377	0.5679	1.283	19.19

**Table 6:** MIC values of copolymer **CP1** and of CP1_1.1-Hgel carried out in triplicate, expressed as µM, computed SI values and MICs of some reference antibiotics.

Strains	CP1 (157,306) ^1^	CP1_1.1-Hgel	SI	Reference Antibiotic
	MIC µM	MIC µM		MIC µM
	***Gram-positive species of genus Enterococcus***			
*E. faecalis* 365 *	0.4	0.4	3.0	366.3 ^2^
*E. faecalis* 450 *	0.8	0.8	1.5	366.3 ^2^
*E. faecalis* 451 *	0.4	0.4	3.0	366.3 ^2^
*E. faecium* 300 *	0.4	0.4	3.0	366.3 ^2^
*E. faecium* 364 *	0.4	0.4	3.0	366.3 ^2^
*E. faecium* 503 *, TR	0.4	0.4	3.0	366.3 ^2^
	***Gram-positive species of genus Staphylococcus***			
*S. aureus* 18 **	0.4	0.4	3.0	386.4 ^3^, 1275.5 ^4^
*S. aureus* 187 **	0.8	0.8	1.5	386.4 ^3^, 1275.5 ^4^
*S. aureus* 195 **	0.4	0.4	3.0	386.4 ^3^, 1275.5 ^4^
*S. epidermidis* 180 ***	0.1	0.1	12	193.2 ^3^, 637.8 ^4^
*S. epidermidis* 181 ***	0.1	0.1	12	193.2 ^3^, 637.8 ^4^
*S. epidermidis* 363 **	0.2	0.2	6	193.2 ^3^, 637.8 ^4^
	***Gram-negative species of Enterobacteriaceae family***			
*E. coli* 461	0.8	0.8	1.5	96.6 ^3^
*E. coli* 462 §	0.8	0.8	1.5	96.6 ^3^
*K. aerogenes* 484CAR	0.8	0.8	1.5	96.6 ^3^
*K. aerogenes* 500 _#_	0.8	0.8	1.5	96.6 ^3^
*K. aerogenes* 501CAR	0.4	0.4	3.0	96.6 ^3^
*K. pneumoniae* 509 _#_	0.8	0.8	1.5	96.6 ^3^
*K. pneumoniae* 520 _#_	0.4	0.4	3.0	96.6 ^3^
	***Non-fermenting Gram-negative species***			
*P. aeruginosa* 1V	0.8	0.8	1.5	76.2 ^5^
*P. aeruginosa* CR	0.8	0.8	1.5	18.5 ^6^
*P. aeruginosa* PY	0.4	0.4	3.0	76.2 ^7^
*A. baumannii* 257	0.8	0.8	1.5	193.2 ^3^

^1^ Mr of copolymer CP1; ^2^ ampicillin; ^3^ ciprofloxacin; ^4^ oxacillin; ^5^ piperacillin tazobactam; ^6^ colistin; ^7^ trimetoprim sulfametoxazole; * denotes vancomycin resistant isolates (VRE); TR = teicoplanin resistant; ** denotes methicillin resistant isolates; *** denotes resistance toward methicillin and linezolid; _#_ denotes carbapenemase (KPC)-producing bacteria; § denotes carbapanemase-(NDM)-producing strains; CAR = carbapanems-resistant isolates not producing β-lactamases; P. aeruginosa, S. maltophylia and A. baumannii are all MDR bacteria; CR = colistin resistant-isolate; PY = pyomelanin-producing strain; 1V = isolated from patient with cystic fibrosis.

**Table 7:** Tendency line polynomial equation, R^2^ value, LD_50_ of CP1, as well as the relative SI ranges.

Sample	Equation	R^2^	LD_50_ (µM)	SI	
				Gram-Positive	Gram-Negative
CP1-based gel	y = 6.0409x^2^ – 46.99x + 98.988	0.9773	1.2	1.5–12	1.5–3

## Data Availability

All data associated to this are comprised in this manuscript.

## Supplementary Materials

The following supporting information can be downloaded at: https://www.mdpi.com/article/10.3390/ijms232315092/s1.
