# Supplementary material for: A Self-Forming Hydrogel from a Bactericidal Copolymer: Synthesis, Characterization, Biological Evaluations and Perspective Applications
Source: Int J Mol Sci. 2022 Dec 1;23(23):15092. doi: 10.3390/ijms232315092 (PMC9741259; doi:10.3390/ijms232315092)
Supplement: Supplementary file 1 [file ijms-23-15092-s001.zip › ijms-2052157-supplementary.pdf]

International Journal of *Molecular Sciences*

MDPI

Supplementary Materials

A Self-Forming Hydrogel from a Bactericidal Copolymer: Synthesis, Characterization, Biological Evaluation and Perspective Applications

Silvana Alfei [1,*], Alessia Zorzoli [2], Danilo Marimpietri [2], Guendalina Zuccari [1], Eleonora Russo [1], Debora Caviglia [3] and Anna Maria Schito [3,*]

[1] Department of Pharmacy, University of Genoa, Viale Cembrano, 16148 Genoa, Italy
[2] Cell Factory, IRCCS Istituto Giannina Gaslini, via Gerolamo Gaslini 5, 16147 Genoa, Italy
[3] Department of Surgical Sciences and Integrated Diagnostics (DISC), University of Genoa, Viale Benedetto XV, 6, 16132 Genova, Italy

* Correspondence: alfei@difar.unige.it (S.A.); amschito@unige.it (A.M.S.); Tel.: +39-010-355-2296 (S.A.)

Section S1

Table S1. Weights of gels at times T_0-T_9.

Time (min)		PD (g)	Swelling ratio (%)
T_0	0	0.1713	0
T_1	1	0.2426	41.6
T_2	3	0.4852	183.2
T_3	5	1.1704	583.2
T_4	10	2.3408	1266.5
T_5	30	2.3980	1299.9
T_6	60	2.6823	1465.8
T_7	90	2.5831	1407.9
T_8	120	2.6800	1464.5
T_9	180	2.6680	1457.5

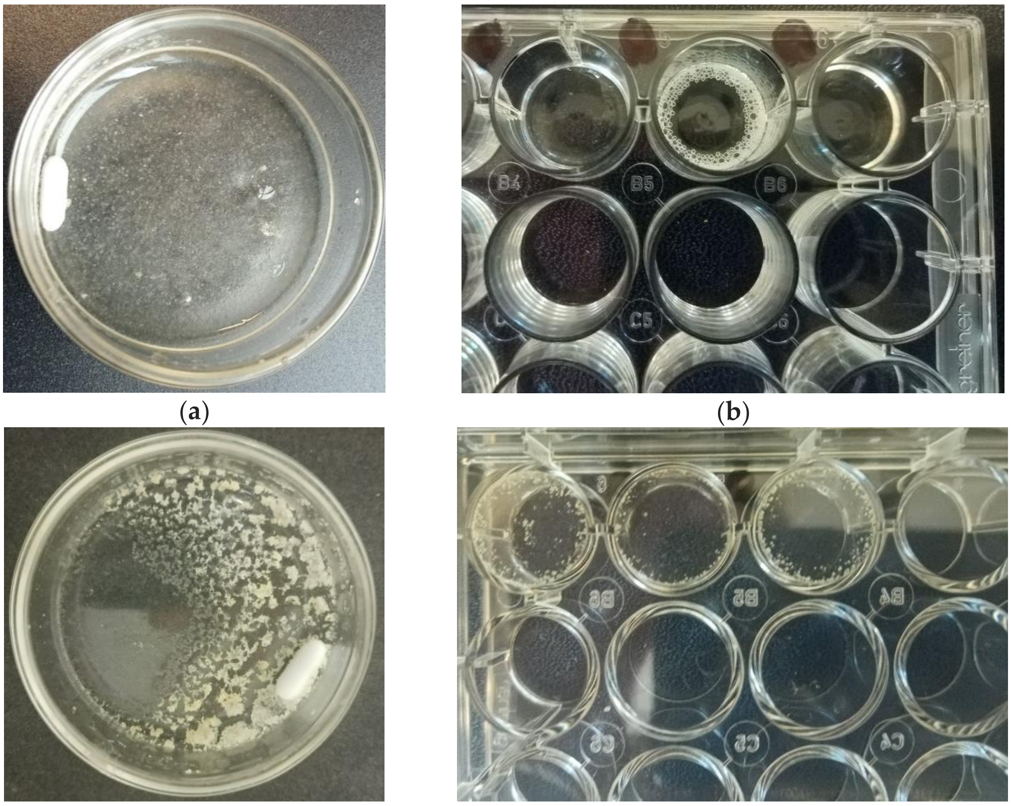

(**a**) (**b**) (**c**) (**d**)

Figure S1. Appearance of CP1_1.1-Hgel at the time t_0 of the experiments carried out to monitor the water loss in time when deposited in the PD (**a**) and in Ws of the 24-wells plate (**b**); appearance of the fully dried samples (time t_x) in the PD (**c**) and in the Ws (**d**).

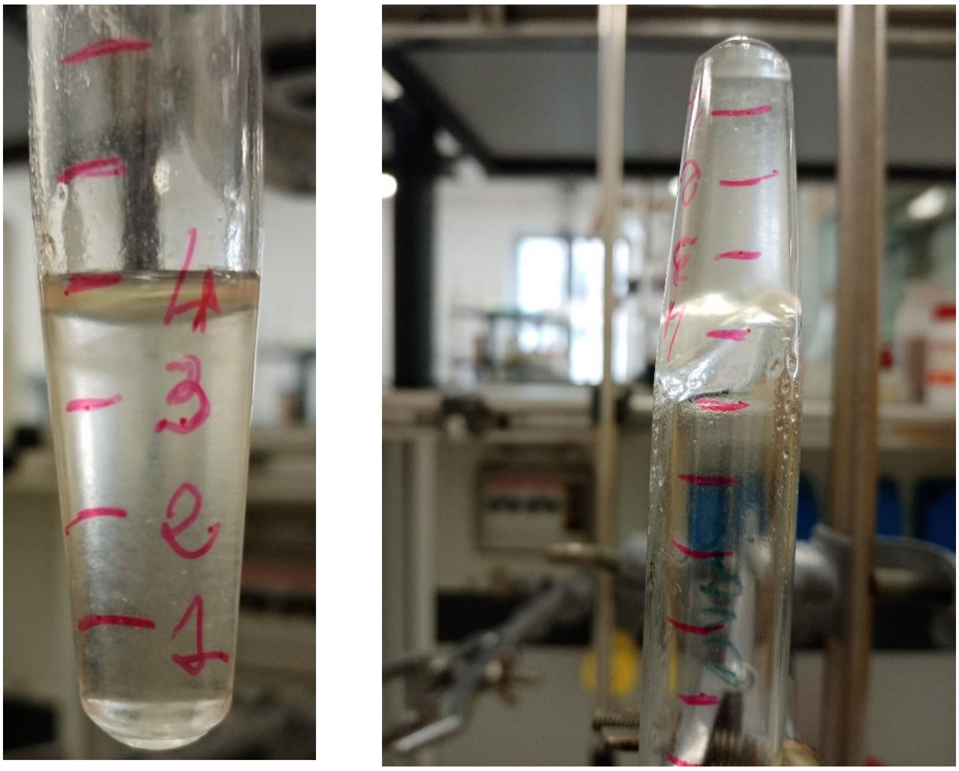

(**a**) (**b**)

Figure S2. Appearance of swollen CP1_1.1-Hgel obtained hydrating the lyophilized gel (1 mL; 91.3 mg) with an excess of 11 mL of water and recovered by centrifugation at 4000 rpm for 25 min, obtaining a CP1-gel at concentration 30.3 mg/mL (3.0 % wt/v) test tube in normal vertical position (**a**); inverted position (**b**).

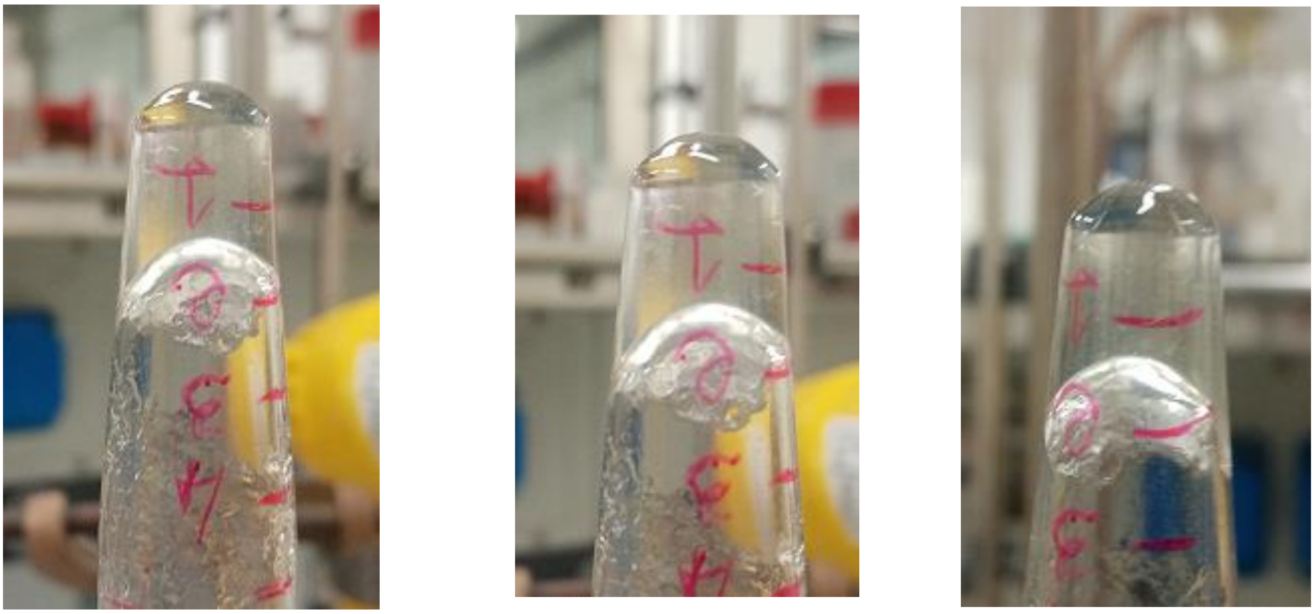

(**a**) (**b**) (**c**)

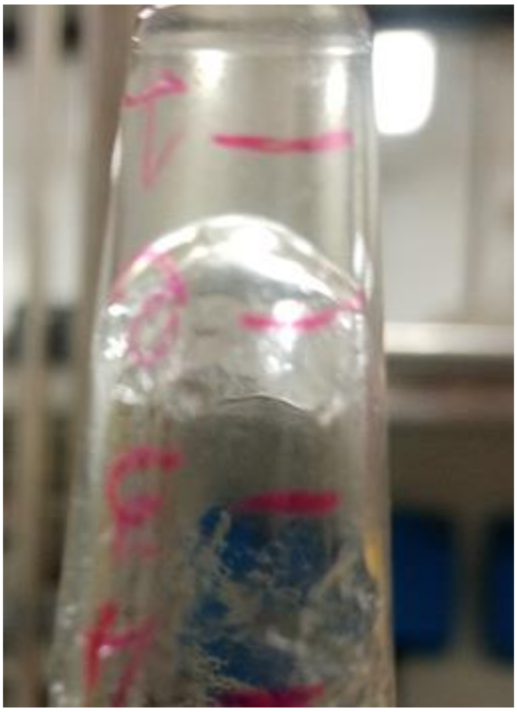

(**d**)

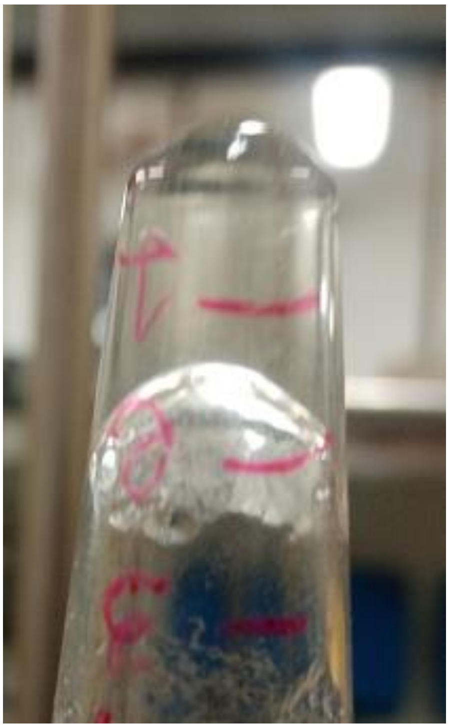

(e)

Figure S3. Appearance of 1 ml of CP1-Hgel (3.0% wt/v) in the inverted position just prepared (**a**), after 1 (**b**), 2 (**c**), 3 (**d**) and 4 months (**e**) from first preparation staying at room temperature.
